# Microalgae as a Nutraceutical Tool to Antagonize the Impairment of Redox Status Induced by SNPs: Implications on Insulin Resistance

**DOI:** 10.3390/biology12030449

**Published:** 2023-03-15

**Authors:** Mattia Melloni, Domenico Sergi, Carolina Simioni, Angelina Passaro, Luca Maria Neri

**Affiliations:** 1 Department of Translational Medicine, University of Ferrara, Via Luigi Borsari 46, 44121 Ferrara, Italy; mattia.melloni@unife.it (M.M.); domenico.sergi@unife.it (D.S.); 2 Department of Life Sciences and Biotechnology, University of Ferrara, Via Fossato di Mortara 70, 44121 Ferrara, Italy; carolina.simioni@unife.it; 3 Laboratory for Technologies of Advanced Therapies (LTTA)—Electron Microscopy Center, University of Ferrara, Via Luigi Borsari 46, 44121 Ferrara, Italy; 4 Medical Department, University Hospital of Ferrara Arcispedale Sant’Anna, Via Aldo Moro 8, 44124 Ferrara, Italy; 5 Research and Innovation Section, University Hospital of Ferrara Arcispedale Sant’Anna, Via Aldo Moro 8, 44124 Ferrara, Italy

**Keywords:** single nucleotide polymorphisms, insulin resistance, microalgae, reactive oxygen species, oxidative stress, antioxidants, nutraceuticals

## Abstract

**Simple Summary:**

Oxidative stress is recognized as one of the pathogenetic mechanisms underpinning insulin resistance, the hallmark of type 2 diabetes. Although oxidative stress can be elicited by unhealthy dietary patterns rich in long-chain saturated fatty acids and sugars, it may be exacerbated in genetically predisposed individuals carrying single nucleotide polymorphisms which dampen the activity of proteins responsible for maintaining redox balance, that is, the balance between oxygen and nitrogen reactive species production and their detoxification. In light of this, this literature review aims at providing an overview of the potential role of microalgae as a source of nutraceuticals able to improve insulin sensitivity by tackling oxidative stress, particularly in the aforementioned individuals. Microalgae represent a source of phenolic compounds, carotenoids, vitamins, and omega-3 polyunsaturated fatty acids which are able to counter oxidative stress, by either modulating intracellular pathways related to inflammation and oxidative stress or by acting as direct reactive oxygen species scavengers. Thus, microalgae represent a promising tool for precise nutritional interventions to tackle oxidative stress and improve insulin sensitivity. Nevertheless, further studies are warranted to confirm whether their supplementation in genetically predisposed individuals may be sufficient to restore redox balance and counter insulin resistance.

**Abstract:**

Microalgae represent a growing innovative source of nutraceuticals such as carotenoids and phenolic compound which are naturally present within these single-celled organisms or can be induced in response to specific growth conditions. The presence of the unfavourable allelic variant in genes involved in the control of oxidative stress, due to one or more SNPs in gene encoding protein involved in the regulation of redox balance, can lead to pathological conditions such as insulin resistance, which, in turn, is directly involved in the pathogenesis of type 2 diabetes mellitus. In this review we provide an overview of the main SNPs in antioxidant genes involved in the promotion of insulin resistance with a focus on the potential role of microalgae-derived antioxidant molecules as novel nutritional tools to mitigate oxidative stress and improve insulin sensitivity.

## 1. Introduction

In genetics, different variability sources have been reported, but among these, single nucleotide polymorphisms (SNPs) are prominent as they represent around 90% of human genetic variation. To be defined as SNPs, the polymorphic alleles arising from the substitution of a single base have to be present in more than 1% of the population. SNPs can occur within the coding sequence of a gene, an intronic or intergenic region, and can consequently affect the amino acid sequence of the resulting protein or modulate the expression of the gene itself, respectively [1]. However, this is not always the case, with not all nucleotide variations leading to an amino acid substitution in the protein encoded by the SNPs affected gene.

On the contrary, however, in some cases, SNPs can act as important predictors of the response to certain specific drugs, susceptibility to certain environmental stressors such as toxins, and the development of some diseases, including metabolic diseases [2]. In this regard, a wide variety of SNPs have been associated with the susceptibility to develop metabolic syndrome (MetS) and type 2 diabetes mellitus (T2DM) [3], which are both underlain by insulin resistance (IR). Insulin resistance is referred to as a blunted response of insulin tissue targets to this hormone leading to impaired glycaemic control, dyslipidemia along with an increased risk of developing cardiovascular disease and certain types of cancer [4,5]. In this regard, insulin resistance impairs glucose homeostasis by negatively affecting glucose uptake and metabolism in metabolically active tissues, namely the skeletal muscle, the adipose tissue, and the liver [6]. In the skeletal muscle, insulin resistance hampers GLUT-4-dependent glucose uptake leading to an impairment in glucose oxidative metabolism and non-oxidative glucose disposal [7]. The same holds true for the adipose tissue, where defective insulin signaling impairs glucose uptake. This occurs in concert with a loss of inhibition of lipolysis leading to an increase in circulating free-fatty acids which further deteriorates insulin sensitivity by promoting lipotoxic lipid accumulation in metabolically active tissues like the liver and the skeletal muscle [8]. Finally, in the liver, the ability of insulin to lower hepatic glucose production is compromised, further fueling circulating hyperglycaemia [9].

To date, a wide array of SNPs has been identified as genetic susceptibility factors for IR, including SNPs in genes encoding proteins directly involved in the insulin signaling pathway such as insulin receptor and in genes responsible for the modulation of insulin sensitivity as described in the next paragraphs. Additionally, other SNPs are able to trigger pathogenetic mechanisms directly involved in hampering insulin signaling, such as oxidative stress [10]. In this regard, several SNPs have been identified in genes encoding enzymes with antioxidant activity and therefore responsible for the maintenance of redox balance [11,12,13].

In particular, aberrant antioxidant genes lead to an imbalance in the cellular redox status underpinned by an increase in reactive oxygen species (ROS) and a decrease in their detoxification. The resulting redox imbalance leads to the disruption of the insulin signaling pathway at different levels, paving the way for the development of IR [14,15].

ROS are reactive molecules due to the presence of at least an unpaired electron in the outermost orbital [16], occur within the cell, and also represent the product of environmental factors such as active and passive smoking, ultraviolet exposure, and psychophysical stress. ROS are unstable molecules that, in order to return to their balanced state, subtract electrons from other nearby atoms, generating new unstable molecules in a chain reaction [17]. In physiological concentrations, ROS have fundamental roles including regulation of gene expression, intracellular communication, defence of the organism from pathogens, and stimulation of immune functions, but the lack of balance can cause damage to cellular components including proteins, lipids, and DNA [18,19,20].

Dietary patterns rich in fat and refined carbohydrates are key in fostering obesity and its comorbidities by triggering inflammation, lipotoxicity, and oxidative stress in metabolically active tissues as well as the hypothalamus [7,21,22]. Considering the deleterious impact of unhealthy dietary patters on oxidative stress, this phenomenon may be exacerbated in individuals carrying SNPs in genes encoding antioxidant proteins, thereby further fuelling insulin resistance [23].

In contrast, following a healthy dietary pattern, like the Mediterranean diet, rich in antioxidant molecules such as carotenoids, vitamins, phenolic compounds, and mono- as well as polyunsaturated fatty acids, coupled with adequate physical activity are essential factors to preserve insulin sensitivity and also by improving redox balance [7,24,25].

In this context, microalgae may represent a novel nutritional tool to counter oxidative stress, particularly due to the fact that these single-celled organisms provide a unique combination of antioxidant molecules [26].

Several microalgae species have already been approved by the major food regulatory authorities for human consumption, and more species are in the process of being approved. Although microalgae also include cyanobacteria, it was chosen not to mention them as they have some limitations in the production of molecules such as carotenoids and vitamins, thus becoming less attractive than green microalgae in regards to their use as functional foods [27,28]. In addition, although it is established that seaweeds and plants approved for human consumption can accumulate the same antioxidant molecules produced in green microalgae, the latter are also inducible and can therefore be used as bioreactors for the synthesis, and bioaccumulation, of specific molecules in response to particular stress stimuli [29]. In addition, microalgae can be grown in tightly controlled environments with the possibility to constantly monitor culture conditions such as temperature, pH, salinity, nutrient concentration, and illumination. This prevents the microalgae cultures from being influenced by environmental conditions as well as chemical and biological contaminations [30]. All these peculiarities make microalgae an excellent novel food and a source of nutraceuticals with multiple applications. In addition, despite their nutritional value, also in terms of antioxidant potential, there are no reports in the literature dissecting the relationship between microalgae antioxidant molecules, oxidative stress, and the effects on insulin sensitivity.

Herein we provide an overview on the main SNPs in antioxidant genes driving insulin resistance and the possible use of microalgae as a source of antioxidant molecules to improve oxidative stress and possibly improve insulin sensitivity, partially compensating the functional impairment induced by SNPs.

## 2. SNPs Promoting IR

Several studies provided evidence that SNPs are associated with cardio–metabolic disorders including obesity, T2DM, and cardiovascular disease [31]. Variants in more than 50 and 80 loci were, respectively, found to be linked with obesity and T2DM [32], and occur in genes that regulate glucose homeostasis, insulin signaling, and energy balance [33]. The mere presence of SNPs may increase the risk of developing metabolic diseases but is not sufficient for their occurrence. Indeed, in the absence of highly penetrant mutations, metabolic disease arises as a consequence of a complex interaction between intrinsic biological factors, including SNPs, and an obesogenic environment characterized by the consumption of highly palatable energy dense foods and physical inactivity. A pivotal metabolic aberration shared between the cardio–metabolic comorbidities linked with obesity is represented by IR [34]. Thus, considering the association between SNPs and impaired cardio–metabolic health, it is not surprising that they may also contribute to the pathogenesis of IR [35]. In this regard, SNPs in genes encoding peroxisome proliferator-activated receptor gamma (PPARγ), insulin receptor substrate (IRS-1), glucokinase regulatory protein (GCKR), insulin-like growth factor I (IGF1), retinoic acid receptor responder 2 (RARRES2), and transcription factor 7 like 2 (TCF7L2) have all been linked with IR. The involvement of these SNPs in the pathogenesis of IR has been extensively reviewed elsewhere [35,36,37].

However, SNPs implicated in the pathogenesis of IR span beyond genes involved in the regulation of insulin signal transduction pathway and metabolic fuel metabolism. In line with this, SNPs in genes involved in regulating redox balance, as detailed below, have also been implicated in the pathogenesis of IR [10].

## 3. The Role of Oxidative Stress in Promoting Insulin Resistance

The insulin receptor is located in plasma membranes of insulin target cells and is characterized by an intermembrane glycoprotein consisting of four subunits: two alpha- and two beta-subunits forming two alpha-beta (α-β) dimers, bound by disulphide bridges [38,39]. Insulin, upon binding to its cognate receptor, induces a conformational change of the β-subunit that includes an extracellular-, a transmembrane-, and an intercellular-domain. Insulin receptor β-subunit has intrinsic tyrosine kinase activity which is activated by insulin-induced conformational change leading to trans autophosphorylation of three tyrosine residues (Tyr 1158, 1162, 1163) in the cytosolic domain of the receptor.

In order to activate the signal transduction pathway, the insulin receptor requires the interaction with the scaffolding proteins IRS1 and IRS2, two members of the family of insulin receptor substrate (IRS), and an adaptor protein called SHC. IRS1 and IRS2 promote propagation and amplification of insulin metabolic signals, whereas the SHC proteins promote and amplify insulin-activated mitogenic signals [40,41].

As a consequence of IRS binding to the insulin receptor, the phosphorylation of multiple tyrosine residues in the COOH-terminal tail of IRS proteins occurs, permitting the recruitment of Src homology 2 domain (SH2) located in the p85 regulatory subunit of phosphoinositide-3-kinase (PI3K) heterodimers. One of the actions of PI3K is to catalyse the synthesis of phosphatidylinositol-3,4,5-triphosphate (PIP3) from phosphatidylinositol-4,5-bisphosphate [42].

The synthesis of PIP3 leads to the amplification of downstream signals through the binding of mainly two effectors: phosphoinositide-dependent kinase 1 (PDK1) and protein-kinase B (Akt). Akt phosphorylation occurs on Thr308 and Ser473 residues by phosphoinositide-dependent kinase 1 (PDK1) and mechanistic target of rapamycin (mTOR), respectively; its phosphorylation acts as a key node in the transduction of insulin signal which occurs again through phosphorylation of downstream targets involved in cellular growth, survival, and metabolism such as glycogen synthase kinase 3, forkhead box O (FOXO) family of transcription factors, BH3-only protein BAD, mTORC1, endothelial nitric oxide synthase [43,44,45,46], and Akt substrate of 160 kDa protein through which vesicle rich in glucose transporter protein 4 is trafficked to plasma membrane [47].

Oxidative stress can promote IR by affecting the insulin pathway at different nodes via the activation of pathways that interfere with insulin signaling [48]. For example, oxidative stress, due to increased ROS production or defects in their detoxification processes, leads to the activation of different serine kinases such as inhibitor kB kinase β (IKK-β), protein kinase C (PKC), mitogen activated protein kinases (MAPK) as p38, extracellular signal-regulated kinase (ERK), c-Jun n-terminal kinases (JNK) which, in turn, can target key actors governing insulin signaling [49].

These kinases can target both the insulin receptor and IRS, depriving them of full signal transduction capacity and consequently leading to an attenuation of insulin response [50]. The phosphorylation of IRS1 on serine residues by JNK and MAPK [47,51,52] also hampers its phosphorylation on tyrosine residues making it more susceptible to proteasome-mediated degradation [49,53].

The activation of JNK by ROS can also occur through the oxidation and inactivation of the JNK-inhibiting MAPK phosphatase and subsequent apoptosis signal-regulating kinase 1 (ASK1) dissociation [54]. Similarly to JNK, as a result of its activation by pro-inflammatory mediators, IKK-β can phosphorylate multiple serine residues on IRS-1 and IRS-2 impairing the metabolic insulin signaling pathway via the inhibition of the tyrosine phosphorylation of IRS-1 mediated by the insulin receptor [51]. IKK-β is also central in the nuclear factor-κB (NF-kB) signaling pathway which also fuels oxidative stress by generating a vicious cycle that bridges together oxidative stress and inflammation [55].

Under basal conditions, NF-kB is sequestered as heterodimer in the cytoplasm by the NF-kB inhibitory protein (IkB) preventing its translocation to the nucleus and the induction of pro-inflammatory genes. However, in response to oxidative stress and pro-inflammatory stimuli, IKK-β phosphorylates IkB, which is ubiquitinated and undergoes proteasomal degradation, releases NF-kB. The NF-kB translocation to the nucleus results in the activation of the expression of genes encoding pro-inflammatory proteins and pro-oxidant enzymes such as inducible nitric oxide synthase (iNOS) and cyclooxygenase 2 (COX-2), promoting a vicious positive loop generated by inflammation and oxidative stress, termed oxinflammation [56,57,58,59].

**Figure 1:**
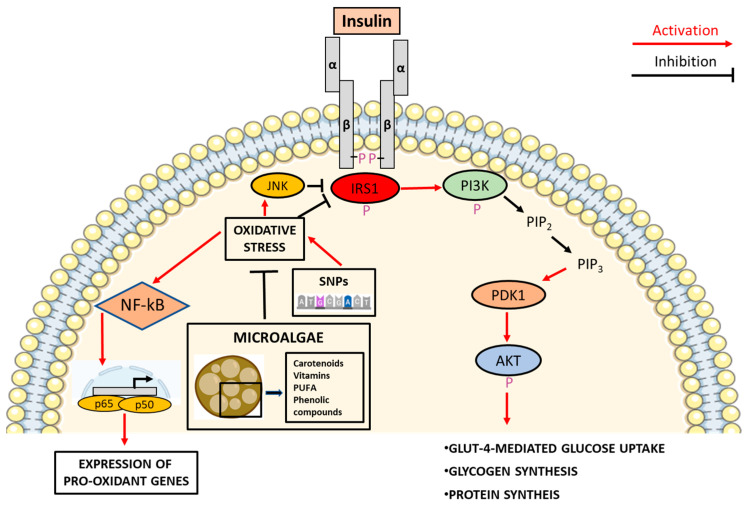
Microalgae mitigate the ability of SNPs in inducing oxidative stress and deregulating insulin signaling.

In conclusion, oxinflammation and the consequent activation of MAPK and IKK-β can, as it is already known, lead to the development of IR by hampering the insulin signaling pathway [60,61] (Figure 1).

## 4. SNPs and Their Involvement in Oxidative Stress

Oxidative stress refers to an imbalance between production of ROS and their removal by cell detoxification systems [62]. Some functional SNPs are present in genes involved in the regulation of redox status, impairing their expression, or altering the functionality of the proteins they encode [63] causing deleterious repercussions on redox balance, with the accumulation of oxygen free radicals and therefore oxidative stress.

Inside the cell, the major oxygen radical producers are the mitochondria, the endoplasmic reticulum, and the peroxisomes [64]. However, mitochondria represent the site where the greatest production of ROS occurs [65].

In the above-cited organelles, given the high production of ROS, enzymes with neutralizing action against free radicals are present, most of which are encoded by genes where numerous SNPs has been found. A list of the main antioxidant genes and the main functional SNPs found in them is reported in the following paragraphs and in Table 1.

### 4.1. Catalase (CAT)

Catalase is an enzyme normally found in all organisms exposed to oxygen and is encoded by the homonymous gene found in humans on chromosome 11p13 [97]. Catalase, one of the most efficient enzymes in the cell, carries out the dismutation reactions of several ROS, in particular H_2_O_2_, which is converted to H_2_O and O_2_. The presence of SNP rs1001179 (C262T) at the promoter region of the *Cat* gene negatively influences the binding of transcription factors and, consequently, the basal transcription and expression of the enzyme [66]. The presence of the T allele, when compared to the C allele, has been associated with reduced enzyme activity, which may contribute to increased levels of ROS and, consequently, to oxidative stress [67]. This SNPs has not only been associated with oxidative stress but also with an increased risk of developing IR [68] and related complications [69].

### 4.2. Superoxide Dismutase (SOD)

Superoxide dismutase (SOD) identifies a class of enzymes containing metal ions in the active site and capable of carrying out redox reactions to dismutate the superoxide anion (O^−2^) into molecular oxygen and hydrogen peroxide. In humans, three forms of SOD are detected: SOD-1, -2, and -3, with location in the cytoplasm, mitochondria, and extracellular space, respectively.

The presence of the functional SNP rs4880 (C47T) in the gene encoding *MnSod* (SOD2) leads to a missense mutation in exon 2 of nuclear chromosome 6q25 [70]. During (cytoplasmic) translation of the sixteenth codon of messenger RNA, the C47T SNP leads to the insertion of a valine instead of an alanine into the forming peptide chain, altering the conformation of mitochondrial targeting sequence and thus impairing the correct delivery of MnSOD to mitochondria [71]. The loss of efficiency in post-translational trafficking of this enzyme results in a reduction of the amount of MnSOD imported into mitochondria and in the diminished neutralizing potential of the superoxide ion [72] leading to IR [73] and poor cardiometabolic health [74].

### 4.3. Glutathione Peroxidase 1 (GPX1)

Glutathione peroxidase (GPx) is the nomenclature assigned to a family of enzymes with peroxidase activity. Eight genes, each encoding a different isoform of glutathione peroxidase (GPX1-8) with different cellular localisation and substrate, have been identified so far. The GPX1 gene located on the nuclear chromosome 3p21.3 encodes for the most representative and abundant enzyme of the family: GPX1 [75]. The presence of the functional SNP rs1050450 on *Gpx1* gene, induces the substitution of a cytosine with a thymine resulting in the replacement of a leucine with a proline at position 198 [76].

This SNP causes the reduction of GPX1 enzyme activity [77], which has also been reported to correlate with the development of IR [78].

### 4.4. Glutathione-S-Transferases (GSTs)

Glutathione s-transferases (*GST*) identify a family of genes encoding proteins essential for the detoxification of endogenous and exogenous metabolite (carcinogens, antitumor drugs, environmental pollutants), including ROS [98]. The glutathione-s-transferases family comprises 16 genes further divided into six subfamilies: *Gst-alpha* (*GstA*), *-mu* (*GstM*), *-omega* (*GstO*), *-pi* (*GstP*), *-theta* (*GstT*), and *-zeta* (*GstZ*) [99].

Several SNPs in the *GstM1*, *T1*, and *P1* genes have been associated with the increase in oxidative stress [100,101,102] and the development of IR as well as T2DM [103,104].

The SNP rs1695 located at the fifth exon of the *GstP1* gene arouses the substitution of a guanine at position 313 with an adenine, causing the substitution of an isoleucine translated from codon 105 with a valine and a decrease in enzymatic activity [79,80,81].

The SNP rs1056806, located at the level of the *GstM1* gene, generates the substitution of a cytosine with a guanine that has been found to predispose to an increased risk of obesity [63,82].

The SNP rs17856199 at the level of the *GstT1* gene modifies the presence of an adenine with a cytosine, generating the substitution of a phenylalanine with a cysteine during the translation of codon 45 [82]. The reported amino acid substitution causes a decrease in hydrophobic interactions in the encoded protein, therefore affecting the functionality of the protein itself and diminishing its detoxifying capacity [83].

### 4.5. Paraoxonase 1 (PON1)

The family of genes encoding paraoxonase proteins encompasses at least three members and includes *Pon1*, *Pon2*, and *Pon3*, which are located in the long arm of chromosome 7 between positions q21.3 and q22.1 [105]. Most of PON1 proteins circulate in the bloodstream bound to high-density lipoproteins (HDL) where they exert antioxidant actions by preventing lipid oxidation and protecting low-density lipoproteins (LDL) from oxidation, thereby contributing to the prevention and cardiovascular disease [105,106,107,108].

Two SNPs in the *Pon1* gene were found to be particularly involved in the promotion of IR: the first one is the functional SNP rs854560 that causes the substitution of an adenine for a thymine in the 55 codon of the mRNA, leading to the substitution of a leucine with a methionine [84]. The reported amino acid substitution was found to reduce *Pon1* transcript levels and, consequently, protein expression, with a reduction in its antioxidant capacity [85,86,87].

Second, the functional SNP rs662 is underpinned by the substitution of an adenine for a guanine at codon 192 with a consequence replacement of a glutamine with an arginine in the resulting protein. This substitution induces a downstream decrease in the activity of the antioxidant enzyme PON1 [84,88]. In line with the effects of other SNPs involved in redox homeostasis, SNPs rs854560 and rs662 have both been associated with IR and T2DM [89,90,91] by a decrease in the antioxidant activity of PON1.

### 4.6. Nuclear Factor-Erythroid 2-Related Factor 2 (Nrf2)

Nuclear factor-erythroid 2-related factor 2 (Nrf2) is an ubiquitous transcription factor encoded by the *Nrf2* gene located on chromosome 2 at position q31 [92]. The functional SNP rs35652124 causes the substitution of a cytosine for a thymine in the promoter region of the *Nrf2* gene at position -653, which impairs NRF2 transcription and, therefore, its protective effects against oxidative stress [92,93].

The functional SNP rs6721961 causes the substitution of a cytosine for an adenine nucleotide upstream of the promoter region of the *Nrf2* gene, precisely 617 bp upstream of the start of the coding region, causing a decreased gene expression of *Nrf2* [96].

Not surprisingly, the SNPs rs35652124 and rs6721961 have both been associated with increased risk for the development of IR and T2DM [13,94,95].

## 5. Microalgae as a Source of Nutraceuticals

Microalgae are unicellular eukaryotic and prokaryotic microorganisms, including cyanobacteria, found in fresh and salt water and able to perform the photosynthesis reactions [30]. Microalgae have been extensively studied in recent years as they are characterized by a high growth rate, high adaptability to different growth conditions, and the ability to accumulate biologically active molecules depending on the metabolism triggered by the conditions in which they are grown. As photosynthetic organisms, autotrophic microalgae do not require organic compounds to support their growth, instead they need water, light as source of energy, CO_2_ as carbon source, and nitrogen and phosphorous as nutrients. However heterotrophic microalgae can use organic compounds, mainly glucose, as a source of energy [109,110]. In light of these specific characteristics, autotrophic microalgae cultivation is environmentally sustainable as they do not require large amounts of water for their growth and are also capable of using atmospheric CO_2_ during photosynthesis, fixing carbon in a plethora of organic molecules while generating oxygen [111]. The ability of microalgae to fix carbon has been quantified as 1.83 kg of CO_2_ per kg of microalgal (bio)mass [112].

Microalgae have pleiotropic applications and as such are being explored for biofuel production in solid, liquid, and gaseous states; biomass production; as well as a functional and nutritional additive [113]. In the nutraceutical field, microalgae are attracting increasing attention as they possess a complete nutritional profile and, most importantly, represent an inducible source of bioactive molecules. In support of their nutritional value as well as safety profile, several microalgae are approved for human consumption by the food and drug administration (FDA) and the European food safety authority (EFSA) also as an ingredient for functional foods such as pasta, yogurt, and biscuits [114].

In terms of their nutritional composition, microalgae are generally rich in proteins, lipids, polysaccharides, pigments, vitamins, and more. Microalgae protein content ranges from 40% to 70% of the dry weight of the cell [115] and remarkably, the nutritional value of microalgae protein has been reported not to be inferior to that of animal proteins [30]. The lipid content of microalgae varies between 20% and 50% of cell dry weight and encompasses triacylglycerols, glycolipids, phytosterols, phospholipids, and some microalgae also contain esterified PUFAs (poly-unsaturated fatty acids) such as arachidonic acid (ARA) and eicosapentaenoic acid (EPA). Microalgae also contain carotenoids, chlorophyll A and B [116], as well as water and lipid soluble vitamins including vitamin A, B1, B2, B6, B12, C, E, biotin, and folic acid [117,118].

Despite their wide nutritional composition, microalgae are united by their antioxidant potential ascribed to a wide array of bioactive molecules able to dampen oxidative stress, with plausible repercussions on insulin sensitivity.

In this regard, a study conducted in a rat model of diet-induced MetS, showed that the inclusion of the microalgae Tetraselmis chuii in the diet provided benefits by promoting the hepatic production of antioxidant enzymes such as GPX, GSH, SOD, and decreasing the expression of the pro-inflammatory genes *Tgfβ1*, *Il-1β*, *Tnf-α*, and *Nf-kb1* [119]. In a further study conducted on equines suffering from MetS, in which there is both a strong accumulation of oxidative stress-related molecules and a decrease in SOD enzyme activity, it was observed that the supplementation with blue-green algae *Arthrospira platensis* resulted in the increase in SOD enzyme activity compared to the control cohort and in a decrease in body weight and insulin resistance [26,120].

To date no clinical trial has investigated the implications of microalgae antioxidant molecules on human metabolic health. Future research is also required to define the digestibility of microalgae and the bioavailability of the antioxidant molecules that they contain, in order to counteract oxidative stress and insulin resistance [119,121].

Thus, in the following paragraphs, the role of microalgae-derived nutraceuticals as putative tools to tackle oxidative stress are described.

## 6. Microalgal Molecules with Antioxidant Action

As described above, microalgae represent natural bioreactors able to upregulate the production of specific molecules in response to exogenous stressors. These molecules include bioactives that can directly or indirectly scavenge free oxygen radicals, thereby countering oxidative stress [122]. In this regard, carotenoids, vitamins, phenolic compounds, and omega-3 fatty acids are the key classes of molecules responsible for the antioxidant properties ascribed to microalgae.

### 6.1. Carotenoids

The health promoting effects attributed to carotenoids are dependent upon their antioxidants and anti-inflammatory properties, in concert with the role of some carotenoids to act as vitamin A precursors [123]. The major dietary sources of carotenoids are represented by fruits, vegetables, legumes, and cereals. However, it must not be overlooked that microalgae also represent a source of carotenoids. Remarkably, compared with the other plant-derived carotenoids, their production in the microalgae is more efficient, cost effective, and not limited by regions and seasons [124].

The carotenoids produced by microalgae, most widely known for their antioxidant properties, are β-carotene, Lutein, astaxanthin, zeaxanthin, fucoxanthin, β-cryptoxanthin, violaxanthin, canthaxanthin, among others [125] (Table 2).

Β-carotene is particularly abundant in *Dunaliella salina* which is considered to be the largest producer of this carotenoid (up to 13% of its biomass), but it is produced also in *Chlorella sorokiniana*, *Nannochloropsis gaditana*, among others [126]. Lutein is a xanthophyll member of the carotenoid family and occurs in both animal as well as plant products like in egg yolk, spinach, corn, and kale [131]. Lutein can be produced by microalgae as was already reported in *Chlorella protothecoides*, *Chlorella sorokiniana*, and *Dunaliella salina* [132]. Astaxanthin can be found in nature mainly in salmon, crustaceans, or krill, but it is also produced by chemical synthesis and in microalgae, mainly *Haematococcus pluvialis* [134] and *Chlorella zofingiensis* [184]. Zeaxanthin is also present in plant and animal products [185] but can be produced by microalgae, particularly in *Nannochloropsis oceanica* as well as in *Chlorella saccharophilia* and *Synechococcus* sp. [137]. Fucoxanthin is the main carotenoid pigment, component of photosynthetic light-harvesting complexes, in marine ecosystems and a member of the xanthophyll family, it can be produced by microalgae and, in particular, *Phaeodactilum tricornutum* [139], but it can also be produced in *Isochrysis galbana*, *Odontella sinensis*, and *Chaetoceros calcitrans* [140]. β-cryptoxanthin is a retinol precursor carotenoid with a chemical structure similar to β-carotene but more polar. Unlike β-carotene, however, β-cryptoxanthin is not present in many foods, and the richest are squash, persimmons, hot peppers, tangerines, and papaya [127]. In addition to the foods listed, this carotenoid is also produced in certain microalgae including *Pandorina morum* and the cyanobacteria *Arthrospira platensis* [142]. Violaxanthin (VX) is a carotenoid found mainly in orange-colored fruit, but it also produced in microalgae, for example, *Chlorella vulgaris*, *Nannochloropsis oceanica*, and *Dunaliella salina* [144,145]. Canthaxanthin (CX) similar to astaxanthin is contained in bacteria, algae, and some fungi [186], but it can also be produced in microalgae, and examples are *Chlorella vulgaris* [146] and *Dactylococcus dissociatus* [147].

**Figure 2:**
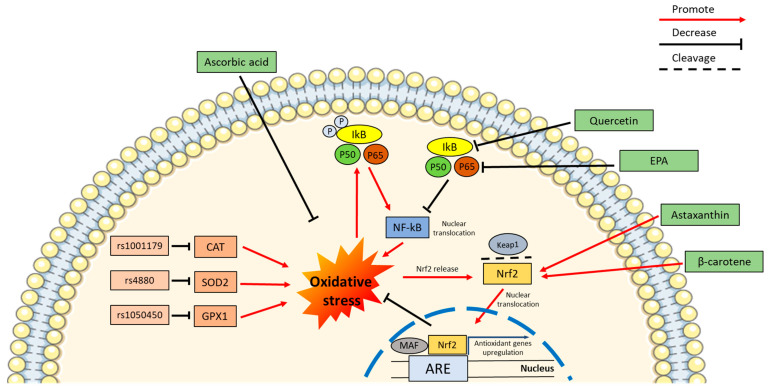
SNPs induced oxidative stress, that could be counteracted by microalgae. Representation of the effect of some polymorphisms on the promotion of oxidative stress leading to a self-maintenance loop via the inflammation pathway. Oxidative stress promotes the phosphorylation of IkB, with the release of NF-kB and its translocation into the nucleus with a consequent expression promotion of pro-inflammatory genes. The molecules produced by microalgae, such as quercetin and EPA, can prevent phosphorylation of IkB and block the self-maintaining loop described above. Other microalgae metabolites such as the carotenoids astaxanthin and β-carotene can promote the translocation of the transcription factor NRF2 from the cytoplasm to the nucleus, as a consequence of the cleavage of its binding to the inhibitor Keap1, promoted by oxidative stress. The NRF2 translocation to the nucleus induced by carotenoids promotes the expression of antioxidant genes through its binding to ARE domains. In addition to the mechanisms mentioned, some microalgae metabolites such as ascorbic acid can reduce the oxidative stress by a direct scavenging activity.

All aforementioned carotenoids exert antioxidant effects via different mechanisms. In particular, they can directly scavenge reactive molecules, particularly singlet molecular oxygen (^1^O_2_) and peroxyl radicals as well as modulate molecular pathways and transcription factors which regulate the expression of genes involved in the ROS detoxification systems. In support of this, β-carotene is able to quench ^1^O_2_ by acquiring the singlet excitation energy, generating triplet-state β-carotene and ground-state oxygen, and subsequently dissipating the energy in the form of heat thereby reconstituting the carotenoid normal energy state [128]. To further support to the antioxidant potential of carotenoids, astaxanthin and β-cryptoxanthin have also been proven to act as ROS scavengers, as demonstrated by different antioxidant activity assay such as ferric reducing antioxidant power (FRAP), trolox equivalent antioxidant capacity (TEAC), oxygen radical absorbance capacity (ORAC), 2,2-diphenyl-1-picrylhydrazyl (DPPH), electron spin resonance spectroscopy (ESR) [187], 2,2′-azino-bis(3-ethylbenzthiazoline-6-sulfonic acid) cation radical (ABTS), and total antioxidant capacity (TAC) [143]. However, as mentioned above, the ability of carotenoids to exploit antioxidant effects, span beyond their capacity to directly scavenge ROS. Indeed, some carotenoids are able to prevent oxidative stress by activating molecular pathways that culminate in the upregulation of antioxidant genes, with these effects being dependent upon NRF2 activation. In the absence of stimuli which perturbate the cell redox balance, the Nrf2 transcription factor is sequestered in the cytoplasm by the Kelch-like ECH associated protein 1 (Keap-1) repressor, which also plays a key role in promoting NRF2 proteasomal degradation [188]. Instead, in response to oxidative stress, the oxidation of specific cysteine residues in the Keap-1 repressor leads to its dissociation from NRF2, which is free to translocate into the nucleus, bind antioxidant responsive element (ARE) regions, and therefore induce the expression of antioxidant genes [189]. Carotenoids have been found to be capable of increasing NRF2-mediated expression of antioxidant genes through the by-products of carotenoids oxidation, which is the result of direct scavenging action and induction of NRF2 translocation to the nucleus. Thus, carotenoids not only directly scavenge ROS, but they also indirectly counteract oxidative stress by inducing genes involved in the cellular machinery responsible for detoxifying ROS [190]. In support of this, supplementation of astaxanthin in subjects affected by T2DM resulted in the upregulation of NRF2 [135]. The induction of this transcription factor in response to carotenoids also holds true for β-carotene [129], lutein [133], zeaxanthin [138], fucoxanthin [141], and β-cryptoxanthin [136] (Figure 2).

The inhibition of the NF-kB signaling represents an additional mechanism underpinning the antioxidant potential of carotenoids. Indeed, carotenoids can hamper the nuclear translocation of the transcription factor NF-kB and the expression of pro-inflammatory molecules as well as genes involved in oxidative stress [130]. It has been hypothesized that carotenoids may exert indirect antioxidant activity by blocking IKK kinase activity and thereby allowing NF-kB sequestration in cytoplasm [191] (Figure 2). Furthermore, in addition to the NF-kB pathway itself, carotenoids can also regulate the action of the MAPKs ERK, JNK, and p38, which have been identified to be involved in the promotion of NF-kB activation [192,193].

Importantly, the ability of carotenoids to dampen oxidative stress and inflammation has also been associated with an improvement in insulin sensitivity, further supporting the role of microalgae as a source of nutraceuticals able to improve metabolic health [194,195,196].

### 6.2. Vitamins

Microalgae are also identified as a source of vitamins. In this regard, vitamins C and E contribute to the antioxidant potential of microalgae [152] (Table 2).

Vitamin C can be obtained from different microalgae such as *Chlorella* whose content can reach 266.67 μg/g Fwt [148], *Nannochloris*, and *Dunaliella* [152]. Vitamin C exerts its antioxidant effects by activating the NRF2 transcription factor as well as by acting as a direct ROS scavenger [149]. Additionally, this vitamin can counter ROS production by inhibiting the NF-kB signaling pathway, which also results in an anti-inflammatory effect [150] (Figure 2).

There is still a lack of consensus on the role of Vitamin C in improving insulin sensitivity. Nevertheless, Shi Lipeng and co-workers performed a meta-analysis of available data from randomized controlled trials on the effect of vitamin C supplementation in patients with T2DM. The result of the analysis, despite some controversies, showed a positive relationship between vitamin C intake, glycaemic control, and insulin sensitivity [151].

With regard to Vitamin E, it is produced, in particular, in *Tetraselmis* (6.32 mg/g DW), *Chlamydomonas* (4 mg/g DW), *Chlorella* (2 mg/g DW), and *Dunaliella* (1.90 mg/g DW) [152]. To the same extent as vitamin C, the antioxidant properties of Vitamin E are also well established and are dependent on its ability to directly scavenge ROS as well as indirectly by inducing *Nrf2* and downregulating the NF-kB pathway [153].

In terms of its metabolic effects, in overweight subjects, vitamin E reduced oxidative stress and improve insulin sensitivity in a plasma concentration-dependent manner [154]. Previous studies have also postulated that the decrease in oxidative stress leads to an improvement in the chemical–physical state of plasma membranes [155] and in this sense, vitamin E has the ability to decrease the curvature of the plasma membrane influencing the activity of enzymes such as protein kinase C and diacylglycerol kinase [156], improving the cell’s response to insulin.

### 6.3. PUFA

Microalgae have also been shown to produce polyunsaturated fatty acids (PUFA) including ω-3 fatty acids. The ability of microalgae to produce ω-3 fatty acids varies depending on the algal species, for examples: C18:3 ω-3 α-linolenic acid (ALA) is mainly produced in *Dunaliella primolecta* [157], *C. vulgaris*, *Chlorococcum amblystomatis*, *Scenedesmus obliquus*, and *Tetraselmis chuii* [158]; C20:5 ω-3 EPA can be produced by *Nannochloropsis oceanica* [164] and *Phaeodactylum tricornutum* [165]; C22:6 ω-3 docosahexaenoic acid (DHA) is produced by *Schizochytrium* sp. [166] and *Isochrysis galbana* [167]; C16:3 ω-3 hexadecatrienoic acid is mainly produced in *C. vulgaris* and C18:4 ω-3 stearidonic acid mainly in *Chlorococcum amblystomatis*, *Scenedesmus obliquus*, and *Tetraselmis chuii* [158] (Table 2).

ω-3 fatty acids have been widely shown to exert anti-inflammatory effects [159,160,161], with the inhibition of inflammation also leading to a decrease in ROS generation. Beside the modulation of inflammation, the ability of ω-3 fatty acids to improve cellular redox status may also depend upon an improvement in mitochondrial function, mitochondrial-ER tethering, and a decrease in ER stress [197]. ω-3 fatty acids possess a modest direct free radical scavenging activity (DHA 28%, EPA 24% compared to the positive control quercetin with 100% scavenging activity) [162], but they mainly reduce oxidative stress indirectly by preserving the IkB inhibitor and thus preventing the translocation of NF-kB into the nucleus [168] (Figure 2).

In addition to the aforementioned mechanisms, ω-3 has also been found to dampen ROS levels by enhancing the expression of genes encoding enzymes with antioxidant activity, such as *Gpx* and heme oxygenase 1 (*Ho-1*), through *Nrf2* gene expression activation [162,163].

In light of this, it is plausible that ω-3 fatty acids may improve IR. Nonetheless, the effects of ω-3 fatty acids on insulin resistance remain controversial with some reports indicating an improvement of insulin sensitivity upon their supplementation [198], whereas other studies fail to confirm this effect [199]. These controversies may be explained by the heterogeneity of the study population, particularly with regard to their ω-3 fatty acid status and the doses as well as the EPA/DHA ratio used in these studies.

### 6.4. Phenolic Compounds

Microalgae can also produce phenolic compounds which have a high ROS scavenging capacity in light of their chemical structure characterized by aromatic ring bearing one or more hydroxyl groups. In keeping with this, microalgae contain several classes of acidic phenols such as gallic, sinapic, and caffeic acids and also flavonoids, including isoflavones, flavanones, and flavonols [200]. Examples of microalgae in which phenolic compounds can be produced are *Chlorella vulgaris*, *Haematococcus pluvial* is, *Diacronema lutheri*, *Phaeodactylum tricornutum*, *Tetraselmis suecica*, and *Porphyridium purpureum* [169] (Table 2).

Phenolic compounds directly counter oxidative stress by acting as electron donors, being oxidized to quinones at the end of the subtraction of the stray electron from the reactive molecules [170,171]. Phenolic compounds may also reduce oxidative stress indirectly through NRF2 activation and subsequent expression of antioxidant genes such as *Gst* but mainly *Ho-1* [172] and via the inhibition of NF-kB pathway [173].

One of the phenolic compounds with insulin sensitizing and antioxidant effects which could be synthetized in microalgae is represented by the flavonol quercetin. In particular, this phenolic compound can be produced by *Tetraselmis suecica* and *Nannochloropsis gaditana* [174]. It was reported that quercetin can directly scavenge free radicals due to the presence of two antioxidant pharmacophores in the molecule, induce the expression of NRF2, and also inhibit ROS-associated inflammation by blocking IKK-β and the nuclear translocation of NF-kB [175,176,177] (Figure 2). Quercetin can be integrated into the diet to stimulate insulin secretion, to protect beta cells from ROS, and to ameliorate the antioxidant defence and inflammation, therefore improving IR [178].

Another phenolic compound which can be synthesized in microalgae is represented by caffeic acid (CA). It can be produced in several microalgae including *Phaeodactylum tricornutum*, *Tetraselmis suecica*, *Nannochloropsis gaditana*, among others [179,180]. CA is capable of directly scavenging radicals such as the superoxide anion with both enzymatic and non-enzymatic reactions [181], to exert stimulatory effect on NRF2 presumably liberating NRF2 from the NRF2-keap1 complex [182], and it can also inhibit the NF-kB pathway, reducing inflammation as well oxidative stress, consequently improving insulin sensitivity [183].

## 7. Conclusions

In conclusion, microalgae represent a novel food rich in nutrients such as proteins, lipids, carbohydrates, vitamins, and minerals, but most importantly, it is an inducible source of bioactive molecules with antioxidant action. Specifically, unlike common seaweeds and plants, functionalized microalgae approved and under approval for human consumption can constitute a valuable nutritional tool for precision nutrition interventions in individuals carrying SNPs which affect their redox status. Thus, particularly in genetically predisposed individuals, the potential of microalgae to restore redox balance may also prevent the development of metabolic aberrations directly related to oxidative stress, such as insulin resistance. However, the fact that these effects may also be elicited by other organisms, such as seaweed, must not be overlooked [201,202]. Nevertheless, studies directly investigating the impact of microalgae intake in individuals with an increased susceptibility to oxidative stress are lacking, particularly those aimed at elucidating the relationship between antioxidant rich microalgae, oxidative stress, and insulin resistance. In addition, the bioavailability of the metabolites produced in green microalgae, the sustainable methods for their extraction and purification for a green economy, and the sensory quality of microalgae formulated food products represent some issues that have to be improved [118]. Despite this, microalgae, in light of their ability to act as bioreactors and accumulate a wide array of antioxidant molecules, remain a promising tool to improve insulin sensitivity by restoring redox balance.

## Figures and Tables

**Table 1:** List of the most representative SNPs involved in promoting IR and modulating oxidative stress.

SNP	Gene Involved	Genetic Variation	SNPs Effect	Reference(s)
rs1001179 (C262T)	*Cat*	Presence of T allele instead of C allele in the *Cat* gene	Increase the levels of ROS and consequently the oxidative stress; Enhance the risk of T2DM development.	[66,67,68,69]
rs4880 (C47T)	*Sod2*	Missense variant in exon 2 of nuclear chromosome 6q25.	Reduce the ability to neutralise the superoxide ion and the risk of IR-related pathologies	[70,71,72,73,74]
rs1050450	*Gpx1*	Substitution of the cytosine nucleotide by the thymine and translation of the amino acid leucine in place of the amino acid proline at position 198	Reduce the GPX1 enzyme activity; Increase the risk of onset of secondary diseases linked to T2DM	[75,76,77,78]
rs1695	*GstP1*	Substitution of the guanine nucleotide at position 313 with the adenine nucleotide	Reduce the enzyme activity	[79,80,81]
rs1056806	*GstM1*	Substitution of nucleotide cytosine to guanine.	Increase the risk of obesity	[63,82]
rs17856199	*GstT1*	Substitution of the adenine nucleotide for the cytosine nucleotide	Reduce the enzyme activity	[82,83]
rs854560	*Pon1*	Substitution of the adenine nucleotide for the thymine nucleotide in the 55 codon of the mRNA	Reduce the *Pon1* transcription levels and its antioxidant capacity	[84,85,86,87]
rs662	*Pon1*	Substitution of the adenine nucleotide for the guanine nucleotide at codon 192	Reduce the activity of the PON1 antioxidant enzyme	[84,88,89,90,91]
rs35652124	*Nrf2*	Substitution of the cytosine nucleotide for the thymine nucleotide in the promoter region of the NRF2 gene at position -214	Increase the oxidative stress and the risk for the onset of IR and T2DM	[13,92,93,94,95]
rs6721961	*Nrf2*	Substitution of the cytosine nucleotide for the adenine nucleotide upstream of the promoter region of the *Nrf2* gene	Reduce the *Nrf2* gene expression and increase the risk for the onset of IR and T2DM	[13,94,95,96]

**Table 2:** List of the main microalgae synthesizing carotenoids, vitamins, PUFA, and phenolic compounds.

Compound	Main Alga/e in Which It Is Found	Compound Role	Molecular Effect	Reference(s)
β-carotene (Carotenoids)	*Dunaliella salina*, *Chlorella sorokiniana*, *Nannochloropsis gaditana*	Anti-oxidant, anti-inflammatory	Increment of NRF2-mediated expression of antioxidant genes; Inhibition of the NF-kB signaling; Inhibition of the expression of cytokines (IL-1 and TNF- α); Phosphorylation inhibition of the of MAPKs.	[126,127,128,129,130]
Lutein (Carotenoids)	*Chlorella protothecoides*, *Chlorella sorokiniana*, *Dunaliella* *salina*	Anti-oxidant, anti-inflammatory	Increment of NRF2-mediated expression of antioxidant genes; Inhibition of the NF-kB signaling.	[130,131,132,133]
Astaxanthin (Carotenoids)	*Haematococcus pluvialis*, *Chlorella zofingiensis*	Anti-oxidant, anti-inflammatory	Increment of NRF2-mediated expression of antioxidant genes; Inhibition of the NF-kB signaling; Inhibition of the expression of cytokines (IL-1 and TNF- α); Phosphorylation inhibition of the of MAPKs.	[130,134,135,136]
Zeaxanthin (Carotenoids)	*Nannochloropsis oceanica*, *Chlorella saccharophilia*, *Synechococcus* sp.	Anti-oxidant, anti-inflammatory	Increment of NRF2-mediated expression of antioxidant genes; Inhibition of the NF-kB signaling; Inhibition of the expression of cytokines (IL-1 and TNF- α); Phosphorylation inhibition of the of MAPKs.	[130,137,138]
Fucoxanthin (Carotenoids)	*Phaeodactilum tricornutum*, *Isochrysis galbana, Odontella sinensis, Chaetoceros calcitrans*	Anti-oxidant, anti-inflammatory	Inhibition of the expression of cytokines (IL-1 and TNF- α); Inhibition of the NF-kB signaling; Phosphorylation inhibition of MAPKs.	[130,139,140,141]
β-cryptoxanthin (Carotenoids)	*Arthrospira platensis*, *Pandorina morum*	Anti-oxidant, anti-inflammatory	Increment of NRF2-mediated expression of antioxidant genes; Inhibition of the NF-kB signaling; Inhibition of the expression of cytokines (IL-1 and TNF- α); Phosphorylation inhibition of the of MAPKs.	[130,136,142,143]
Violaxanthin (VX) (Carotenoids)	*Chlorella vulgaris*, *Nannochloropsis oceanica*, *Dunaliella salina*	Anti-oxidant, anti-inflammatory	Increment of NRF2-mediated expression of antioxidant genes; Inhibition of the NF-kB signaling; Inhibition of the expression of cytokines (IL-1 and TNF- α); Phosphorylation inhibition of the of MAPKs.	[130,144,145]
Canthaxanthin (CX) (Carotenoids)	*Chlorella vulgaris*, *Dactylococcus dissociatus*	Anti-oxidant, anti-inflammatory	Increment of NRF2-mediated expression of antioxidant genes; Inhibition of the NF-kB signaling; Inhibition of the expression of cytokines (IL-1 and TNF-α); Phosphorylation inhibition of the of MAPKs.	[130,146,147]
Vitamin C (Vitamins)	*Chlorella vulgaris*, *Nannochloris* *Oceanica*, *Dunaliella* *salina*	Anti-oxidant, anti-inflammatory	Activation of the NRF2 transcription factor; Inhibition of the NF-kB signaling pathway.	[148,149,150,151]
Vitamin E (Vitamins)	*Chlorella vulgaris,* *Dunaliella* *Salina*, *Tetraselmis Chlamydomonas*	Anti-oxidant, anti-inflammatory	Inactivation of Keap-1 and subsequent release of NRF2 transcription factor; Downregulation of the IkB-NF-kB pathway.	[152,153,154,155,156]
C18:3 ω-3 ALA (PUFA)	*Dunaliella primolecta*, *Chlorella vulgaris*, *Chlorococcum amblystomatis,* *Scenedesmus obliquus,* *Tetraselmis chui*	Anti-oxidant, anti-inflammatory	Preservation of the IkB inhibition, thus preventing the translocation of NF-kB into the nucleus; Activation of NRF2.	[157,158,159,160,161,162,163]
C20:5 ω-3 EPA (PUFA)	*Nannochloropsis oceanica*, *Phaeodactylum tricornutum*	Anti-oxidant, anti-inflammatory	Preservation of the IkB inhibition, thus preventing the translocation of NF-kB into the nucleus; Activation of NRF2.	[159,160,161,162,163,164,165]
C22:6 ω-3 Docosahexaenoic acid (DHA) (PUFA)	*Schizochytrium* sp., *Isochrysis galbana*	Anti-oxidant, anti-inflammatory	Preservation of the IkB inhibition, thus preventing the translocation of NF-kB into the nucleus; Activation of NRF2.	[159,160,161,162,166,167,168]
C16:3 ω-3 Hexadecatrienoic acid (PUFA)	*Chlorella vulgaris*	Anti-oxidant, anti-inflammatory	Preservation of the IkB inhibition, thus preventing the translocation of NF-kB into the nucleus; Activation of NRF2.	[158,161,163]
C18:4 ω-3 Stearidonic acid (PUFA)	*Chlorococcum amblystomatis*, *Scenedesmus obliquus*, *Tetraselmis chui*	Anti-oxidant, anti-inflammatory	Preservation of the IkB inhibition, thus preventing the translocation of NF-kB into the nucleus; Activation of NRF2.	[158,161,163]
Quercetin (Flavonol)	*Tetraselmis suecica*, *Nannochloropsis gaditana*	Anti-oxidant, anti-inflammatory	NRF2 activation and subsequent expression of antioxidant genes; Inhibition of NF-kB pathway by blocking IKK-β.	[169,170,171,172,173,174,175,176,177,178]
Caffeic acid (Acid phenol)	*Phaeodactylum tricornutum*, *Tetraselmis suecica*, *Nannochloropsis gaditana*, *Arthrospira platensis*	Anti-oxidant, anti-inflammatory	NRF2 activation and subsequent expression of antioxidant genes; Inhibition of NF-kB pathway.	[169,170,171,172,173,179,180,181,182,183]

## Data Availability

Not applicable.
